# HLA Class II Immunogenetic Profiles Shape Psychosis Outcomes in Cannabis Users: DRB5/DRB1*16 Vulnerability and DRB4/DRB3-Linked Protection, Particularly Against Schizophrenia

**DOI:** 10.3390/neurosci7030056

**Published:** 2026-05-07

**Authors:** Andrei Buciuta, Horia George Coman, Bogdan Nemeș, Mihaela Elvira Cimpianu, Radu Oroian, Mihaela Laura Vică Matei, Horea-Vladi Matei

**Affiliations:** 1 Department of Cellular and Molecular Biology, Iuliu Haṭieganu University of Medicine and Pharmacy, 400006 Cluj-Napoca, Romania; 2 Department of Medical Psychology and Psychiatry, Iuliu Haṭieganu University of Medicine and Pharmacy, 400012 Cluj-Napoca, Romania; 3 Department of Nursing, “1 Decembrie 1918” University of Alba Iulia, 510009 Alba Iulia, Romania; 4 Zalau County Emergency Hospital, 450129 Zalău, Romania

**Keywords:** cannabis, psychotic disorders, schizophrenia, HLA class II, immunogenetics, genetic susceptibility

## Abstract

Background: Only a subset of cannabis users develop persistent psychosis, implying that genetic vulnerability modulates risk. HLA-DR/DQ variation is a strong non-dopaminergic risk locus for schizophrenia, but its role in cannabis-related psychosis is unclear. Methods: We studied 296 cannabis users from Romanian psychiatric services, grouped as non-psychosis (0), non-schizophrenia psychosis (1) and schizophrenia (2). High-resolution HLA-DRB1, DRB3/4/5 and inferred DRB1-DQB1 haplotypes were tested using Fisher’s exact tests with FDR correction in a universal contrast (0 vs. 1+2) and 0-1-2 pairwise comparisons, with Firth logistic regression and resampling as supportive analyses. Results: In the universal analysis, DRB1*16, DRB5 and the DRB1*16-DQB1*05 haplotype were associated with roughly two- to threefold higher odds of psychosis, whereas DRB1*07 and DRB4/DRB3 paralogs showed protective effects or trends. In the 0-1-2 contrasts, DRB1*16 was enriched in non-schizophrenia psychosis. DRB4/DRB3 paralogs were under-represented in schizophrenia relative to both cannabis users without psychosis and those with non-schizophrenia psychosis, suggesting a schizophrenia-specific protective association. Firth models supported effect directions but were underpowered. Conclusions: HLA class II immunogenetic background may modify psychosis risk among cannabis users: DRB5/DRB1*16-containing backgrounds were associated with increased vulnerability, whereas DRB4/DRB3 paralogs were associated with reduced schizophrenia risk in this cohort. These findings are hypothesis-generating, do not establish causality, and warrant replication in larger cohorts.

## 1. Introduction

Schizophrenia and other psychotic disorders arise from a complex interplay between genetic and environmental factors. Among the environmental exposures implicated, cannabis use shows one of the most consistent associations [1]. Epidemiological and genetic studies indicate that heavy or early cannabis use increases the risk of psychotic disorders [2,3], yet only a minority of exposed individuals develop persistent psychosis, suggesting that biological susceptibility modulates individual responses to cannabis [4]. Systematic reviews have highlighted susceptibility genes in the dopaminergic system, including COMT, DRD2, DAT and AKT1 [5,6], which influence how the dopaminergic system responds to exogenous cannabinoids, but these variants explain only a small proportion of the phenotypic variance and therefore do not fully account for the pathogenesis of psychotic disorders [7].

Beyond dopaminergic mechanisms and cannabis exposure, immune dysregulation and pathogen-related effects have been implicated as major contributors to psychosis [8], bringing the HLA region into focus as a key candidate [9]. HLA genes, which encode major histocompatibility complex class I and II molecules that present antigens to T cells and initiate adaptive immune responses, are strongly associated with numerous autoimmune and infectious diseases. Genome-wide association studies have identified the HLA region on chromosome 6p21 as one of the strongest non-dopaminergic susceptibility loci for schizophrenia [10], and multiple case–control and family-based studies have linked HLA-DRB1 and HLA-DQB1 polymorphisms to schizophrenia risk, with some alleles (e.g., DRB1*03, DRB1*04) conferring increased susceptibility and others (e.g., DRB1*13, DRB1*14) appearing protective [11,12]. Recent work further suggests that HLA variation may influence schizophrenia risk by modulating susceptibility to neurotropic herpesviruses and by regulating microglial HLA-DR expression, thereby affecting neuroinflammation and synaptic processes [13,14].

Despite the established role of HLA in schizophrenia and the well-documented contribution of cannabis exposure to psychosis risk, the immunogenetic basis of cannabis-related psychotic disorders remains poorly defined. Most gene–cannabis interaction studies have focused on dopaminergic and endocannabinoid pathways [7,15], with limited attention to HLA class II genes such as HLA-DRB1, HLA-DQB1 and HLA-DRB3/4/5 in cannabis-related psychosis. At the same time, emerging data on substance use disorders suggest that HLA-based risk profiles may differ between drug classes, and cannabis use disorder may have a distinct HLA signature compared with other addictions, supporting a potential role for immunogenetics in cannabis-related phenotypes [16,17,18]. Investigating HLA-DR/DQ variation in well-characterized cohorts of cannabis users with and without psychosis may help clarify whether specific immunogenetic backgrounds modify the likelihood of developing psychotic disorders, including schizophrenia, after cannabis exposure.

In the present work, we focused specifically on high-resolution HLA-DRB1/DQB1 and DRB3/DRB4/DRB5 genotyping because class II loci show robust, replicated associations with schizophrenia and related immune-mediated brain disorders, and because the funded genotyping panel for this doctoral project covered only a limited set of HLA markers. HLA class I loci (such as HLA-B) and complement/MHC III region markers (e.g., C4) were therefore beyond the scope of the current study and represent important targets for future work.

## 2. Materials and Methods

### 2.1. Study Design and Participants

We conducted a cross-sectional study of individuals with a history of cannabis use recruited from psychiatric services and from other clinical/legal settings. Cannabis users without psychosis were recruited from multiple sources, including Psychiatry III, Emergency County Hospital, Cluj, Eurosan Clinic, Cluj-Napoca, and the Institute of Forensic Medicine, Cluj, whereas cannabis users with psychotic disorders were recruited exclusively from psychiatric services. No psychotic individuals were enrolled from the forensic/other clinical stream, because patients with manifest psychosis in those settings are routinely referred to psychiatric care rather than retained in the referring service.

Participants were divided into three groups: (0) cannabis users without psychosis, (1) cannabis users with non-schizophrenia psychotic disorders, and (2) cannabis users with schizophrenia. For the main (“universal”) analysis, groups 1 and 2 were combined as cannabis users with psychosis. A secondary analysis used only groups 0, 1 and 2 to compare no psychosis, non-schizophrenia psychosis and schizophrenia. Diagnoses and psychosis status were established by qualified psychiatrists using standard DSM-5 criteria based on clinical interviews and chart reviews. All participants provided written informed consent to participate in the study and to the use of their blood samples for genetic analyses, including HLA typing. The study was conducted in accordance with the Declaration of Helsinki, and the protocol was approved by the Ethics Committee (AVZ38/14.12.2021).

All participants were recruited from psychiatric and related services within a single geographic region in Romania. However, ancestry-informative genetic markers were not collected, and we did not perform formal ancestry adjustment. Given the strong population specificity of HLA alleles, residual confounding by ancestry cannot be excluded, even in this geographically restricted cohort.

In this study, “cannabis users” were defined as individuals with a documented history of cannabis consumption established by psychiatric interview and review of clinical records, irrespective of current use status. The primary inclusion criterion on the exposure side was a diagnosis of cannabis use, abuse or dependence recorded in the treating service, rather than a specific quantitative threshold of recent consumption. However, based on clinical interviews and chart reviews, the participants included in this study were identified as regular consumers, defined as using cannabis at least weekly. Detailed quantitative measures of cannabis exposure beyond this baseline frequency, such as exact dose, cumulative lifetime duration, and age at first use, were not systematically collected with a standardized instrument and were therefore unavailable for analysis. As a result, our comparisons focus on psychosis outcomes among clinically identified, regular cannabis users, rather than on dose–response relationships across finer gradients of cannabis exposure. Accordingly, the present data do not allow any inference about dose–response effects or specific thresholds of cannabis consumption related to psychosis risk.

### 2.2. HLA Genotyping

Peripheral venous blood was collected into 2 mL EDTA tubes and stored at −20 °C until processing. Genomic DNA was isolated from whole blood using the Maxwell RSC Blood DNA Kit (Promega, Madison, WI, USA), following the manufacturer’s protocol. Briefly, 30 µL of proteinase K was added to 300 µL of blood in an incubation tube, followed by 300 µL of lysis buffer, and the mixture was vortexed for 10 s. The samples were then incubated at 56 °C for 20 min and transferred to the Maxwell RSC cartridges, which were processed on the automated extraction platform. After extraction, DNA was eluted into the supplied buffer. DNA yield and purity were checked spectrophotometrically, and only samples meeting predefined quality criteria were used for HLA typing.

High-resolution HLA class II genotyping focused on HLA-DRB1, HLA-DQB1 and the DRB3/DRB4/DRB5 paralogs. Genotyping was performed using a commercial sequence-specific priming PCR (SSP-PCR) assay (FluoGene DR/DQ kit, Inno-train Diagnostik GmbH, Kronberg, Germany), according to the manufacturer’s instructions. Genomic DNA was diluted in RNase/DNase-free water and mixed with the provided fluorescent master mix to obtain a working concentration of approximately 1 ng/µL, and 15 µL of this mixture was dispensed into each well of the pre-loaded HLA-DR/DQ PCR plate. Amplification was carried out on a thermal cycler with an initial denaturation step at 95 °C for 2 min, followed by 40 cycles of 15 s at 95 °C and 60 s at 60 °C, and a final cooling step at 20 °C for 3 min. HLA-DRB1, HLA-DQB1 and DRB3/DRB4/DRB5 genotypes were assigned using the FluoVista fluorescence reader and the accompanying interpretation software (Inno-train Diagnostik GmbH).

For analysis, DRB1 and DQB1 allele groups were converted into categorical markers (e.g., DRB1*16, DQB1*05), and DRB3, DRB4 and DRB5 were coded as paralog-specific presence/absence indicators. DRB1-DQB1 haplotypes were inferred at the individual level by enumerating all phase-compatible combinations of the observed DRB1 and DQB1 alleles in each genotype (e.g., DRB1*16-DQB1*05, DRB1*15-DQB1*06). Each distinct DRB1-DQB1 combination was then coded as a binary presence/absence marker and analyzed using the same association pipeline as for single-locus alleles. This inference was deterministic and did not use a statistical phasing algorithm. It assumes that the observed DRB1-DQB1 allele pairs approximate common haplotypic configurations, but phase may be uncertain for rare combinations, so haplotype-level results should be viewed as exploratory signals at the level of broader DRB1-DQB1 backgrounds.

### 2.3. Group Contrasts

For the universal analysis, we contrasted cannabis users with psychosis (groups 1+2) against cannabis users without psychosis (group 0). In the 0-1-2 analysis, we examined three pairwise comparisons: 0 vs. 1 (no psychosis vs. non-schizophrenia psychosis), 0 vs. 2 (no psychosis vs. schizophrenia) and 1 vs. 2 (non-schizophrenia psychosis vs. schizophrenia). Group codes (0, 1, 2) were treated as fixed categories and not altered.

### 2.4. Statistical Analysis

Statistical analysis was performed within the R computing environment (version 4.5.0). Data processing, visualization, and modeling were facilitated by the dplyr, tidyr, ggplot2, scales, and logistf packages. 

Univariate associations between each HLA marker and psychosis status were tested with Fisher’s exact test in 2 × 2 tables, which is appropriate for modest sample size and rare alleles. For each contrast, we constructed presence/absence tables for all observed DRB1 alleles, DRB3/4/5 paralogs and DRB1-DQB1 haplotypes. To obtain stable odds ratios when cell counts were small or zero, we applied the Haldane–Anscombe correction (adding 0.5 to each cell) before calculating odds ratios and approximate 95% confidence intervals. Multiple testing within each contrast was addressed using the Benjamini–Hochberg false discovery rate (FDR) procedure applied to the complete set of *p*-values for all tested HLA markers in that contrast; *p*-values were not pre-filtered before FDR correction. Markers with *p* < 0.05 were considered statistically significant; those with 0.05 ≤ *p* < 0.15 were treated as trends; and markers with *p* ≥ 0.15 were not pursued further. Reporting focused on markers reaching at least trend level, with odds ratios, confidence intervals, *p*-values and FDR-adjusted q-values. This trend category was used solely to flag hypothesis-generating signals; such associations remain highly sensitive to sampling variability and should not be taken as evidence of a biological effect in the absence of independent replication.

To examine whether the main HLA effects persisted when considered jointly, we fitted Firth penalized logistic regression models to reduce small-sample bias and address separation. In the universal analysis, models included DRB5, DRB1*16 and, in a secondary specification, a DRB3/DRB4 paralog indicator, comparing psychosis (1+2) versus no psychosis (0). In the 0-1-2 subset, an analogous model compared group 0 with groups 1+2, including indicators for DRB5, DRB3/DRB4 and DRB1*14. These multivariable models were specified in an exploratory manner using a small set of markers that showed the strongest and most consistent univariate associations and were therefore intended to assess robustness and effect direction rather than to provide independent, fully adjusted hypothesis tests.

Given the modest sample size, we did not include the full set of demographic, clinical and environmental covariates in these models, so residual confounding by unmodeled factors, including other substance use and clinical characteristics, is likely. The multivariable results should therefore be interpreted as exploratory and hypothesis-generating rather than as evidence for causally independent HLA effects.

For the strongest signal, we evaluated robustness using permutation and bootstrapping. The empirical *p*-value was obtained by repeatedly permuting group labels and counting how often the Fisher *p*-value for DRB5 was at least as small as observed. A bootstrap 95% confidence interval for the DRB5 odds ratio was derived from resampling individuals with replacement and recalculating the odds ratio across resamples. All analyses were performed in R using custom scripts applied to the exported clinical and HLA datasets.

## 3. Results

### 3.1. Definitions

We analyzed 296 cannabis users divided into three cohorts: those without psychosis (cohort 0), those with non-schizophrenia psychosis (cohort 1) and those with schizophrenia (cohort 2). The primary (“universal”) analysis compared cannabis users without psychosis (0) to those with psychosis (1+2). A secondary 0-1-2 analysis used only cohorts 0, 1 and 2 to allow pairwise comparisons between no psychosis, non-schizophrenia psychosis and schizophrenia.

The sociodemographic characteristics of the study cohorts are shown in Table 1.

### 3.2. Universal Analysis: Groups 0 vs. 1+2

In the universal contrast, several HLA class II markers were associated with psychosis after FDR correction. DRB1*16 showed a significant association, as did the DRB3/4/5 paralog marker corresponding to HLA-DRB5, and the DRB1*16-DQB1*05. DRB1*07 showed a protective effect, while DRB4 and DRB3 paralogs had odds ratios below 1 with trend-level *p*-values (Table 2).

A Firth penalized logistic model including DRB5 and DRB1*16 supported these directions (OR 1.95 for DRB5, *p* = 0.074; OR 2.39 for DRB1*16, *p* = 0.057), and adding DRB4 did not materially change the estimates; DRB4 itself was not significant (OR 0.91, *p* = 0.85). For DRB5, the Fisher *p*-value was 0.0022, the permutation *p*-value 0.0012, and the bootstrap odds ratio 2.33 (95% CI 1.39–3.75), indicating a robust association.

### 3.3. The 0-1-2 Analysis: Pairwise Comparisons

In the 0-1 comparison (cannabis users without psychosis vs. cannabis users with non-schizophrenia psychosis), DRB1*16 was enriched in the psychosis group. The DRB1*15-DQB1*06 haplotype showed a protective trend but did not meet significance thresholds (Table 3).

In the 0-2 comparison (no psychosis vs. schizophrenia), the DRB4/DRB3 paralog positivity was strongly under-represented in schizophrenia and was therefore consistent with a protective association in this cohort, whereas DRB5 was positively associated with schizophrenia. DRB1*03 showed a large but imprecise odds ratio and was considered exploratory (Table 4).

In the 1-2 comparison (non-schizophrenia psychosis vs. schizophrenia), DRB4 paralog positivity again was under-represented in schizophrenia, reinforcing its protective role. DRB1*16 was relatively less frequent in schizophrenia than in non-schizophrenia psychosis, suggesting differing HLA-DRB1 distributions between psychosis subtypes. DRB1*08 and DRB1*03 showed large risk-direction odds ratios but wide confidence intervals and only trend-level q-values (Table 5).

A further Firth model in the 0-1-2 subset (0 vs. 1+2) including DRB5, DRB4/DRB3 and DRB1*14 yielded odds ratios in the expected directions but with very wide confidence intervals and non-significant *p*-values, and was therefore treated as supportive rather than primary evidence.

## 4. Discussion

In this immunogenetic study of individuals with a history of cannabis use, we found consistent associations between HLA class II profiles and psychotic outcomes [11,13]. DRB5/DRB1*16-containing backgrounds were linked to roughly two- to threefold higher odds of psychotic disorders, whereas DRB4/DRB3 paralogs were associated with a lower likelihood of schizophrenia within the exposed group. Together, these observations extend the gene–cannabis literature, which has focused largely on dopaminergic and endocannabinoid pathways and on polygenic risk scores, by highlighting immune-related variation as an additional layer of vulnerability and resilience in cannabis-related psychosis [19].

Mechanistic interpretations linking HLA class II variation to viral susceptibility, autoimmunity or neuroinflammation remain hypothetical in this context, as the present cross-sectional genetic data do not include functional immune or pathogen measures and cannot support causal inferences.

In the primary comparison of cannabis users with psychosis versus those without psychosis, DRB1*16 and the DRB3/4/5 marker corresponding to HLA-DRB5 both showed robust associations with psychosis, with FDR-corrected q-values around 0.007 and odds ratios in the two- to threefold range. The DRB1*16-DQB1*05 haplotype further strengthened this signal, with an odds ratio above 3 and FDR-corrected significance. Permutation and bootstrap analyses confirmed that the DRB5 association was unlikely to reflect chance, yielding a permutation *p*-value near 0.001 and a bootstrapped odds ratio around 2.3 with a reasonably narrow confidence interval. These results suggest that DRB5- and DRB1*16-containing haplotypes define a class II background that increases susceptibility to psychosis in cannabis-exposed individuals, consistent with the established role of the HLA-DR region in immune regulation and its prior implication in schizophrenia [11,20].

It is important to note that DRB1*16, the DRB5 paralog marker and the DRB1*16-DQB1*05 haplotype are not independent signals, but rather reflect a shared HLA class II background. DRB5 is typically carried on DRB1*15/16 haplotypes, and DRB1*16-DQB1*05 represents a specific configuration within this extended region, so the observed associations likely arise from correlated variation across these markers rather than from three separate, additive effects. Our analyses therefore identify a DRB1*16/DRB5-containing haplotypic background associated with increased psychosis risk in cannabis users, without resolving which specific locus or combination is causally responsible.

The 0-1-2 analysis refined these observations and highlighted differential effects across psychosis subtypes. DRB1*16 was clearly enriched in non-schizophrenia psychosis compared with cannabis users without psychosis, indicating that it contributes specifically to psychotic outcomes rather than to cannabis use per se. In contrast, DRB4/DRB3 paralog positivity was strongly and reproducibly protective for schizophrenia: DRB4/DRB3 backgrounds were substantially under-represented in schizophrenia compared both with non-psychotic cannabis users and with cannabis users who had non-schizophrenia psychoses. This pattern aligns with immunogenetic studies suggesting that certain HLA-DR/DQ configurations confer protection against schizophrenia, potentially via more efficient viral control or reduced autoimmune activity [11,13]. The combination of DRB5-associated risk and DRB4/DRB3-associated protection supports a model in which distinct class II backgrounds modulate the trajectory from cannabis exposure to schizophrenia versus other psychotic outcomes.

DRB1*16 showed a variable pattern across contrasts: more frequent in psychotic versus non-psychotic cannabis users and in non-schizophrenia psychosis versus no psychosis, but relatively less frequent in schizophrenia than in non-schizophrenia psychosis. This suggests that DRB1*16 increases overall vulnerability to psychotic disorders in cannabis users but is more strongly linked to non-schizophrenia psychoses than to schizophrenia. Similar diagnostic “shifts” in risk have been described for other variants, which raise the general risk of psychosis while influencing the specific diagnostic profile. In our sample, DRB1*16 appears to mark a psychosis-prone subset of cannabis-using patients who are preferentially represented in non-schizophrenia psychotic diagnoses, whereas DRB4/DRB3 define a subset with reduced likelihood of schizophrenia.

Multivariable Firth logistic models, constructed on a restricted set of markers pre-selected from the univariate results, did not yield individually significant effects for DRB5, DRB1*16 or DRB4/DRB3, but their odds ratios remained in the directions indicated by the Fisher tests. The wide confidence intervals and non-significant *p*-values likely reflect modest sample size, low allele frequencies and strong linkage within the HLA region, as well as the fact that model covariates were chosen based on univariate signals, which may introduce selection bias. Given these constraints, the multivariable models are best regarded as exploratory and supportive of effect direction rather than as definitive evidence of independent effects.

Our findings complement existing work on genetic modulation of cannabis-related psychosis, which has primarily implicated COMT, DRD2, AKT1, endocannabinoid receptors and schizophrenia polygenic risk scores [5,19]. Larger studies have shown that individuals with higher schizophrenia genetic load and certain dopaminergic genotypes are more likely to develop psychotic disorders in the context of cannabis use [16,21,22]. By adding HLA-DRB5, DRB1*16 and DRB4/DRB3 to this picture, the present findings are consistent with immune-related genetic variation acting as a potential modifier of cannabis-associated psychosis risk rather than as a standalone determinant. In a broader framework, dopaminergic genes may influence neurochemical sensitivity to cannabis, polygenic risk may reflect distributed neurodevelopmental liability, and HLA-related immunogenetic backgrounds may interact with cannabis-induced stressors, infections or inflammatory processes to shape who transitions from cannabis use to persistent psychotic illness, a hypothesis that requires direct mechanistic testing [15,23].

Several limitations temper these conclusions and support an exploratory interpretation. The overall sample was moderate and the schizophrenia subgroup small, which led to wide confidence intervals and limited statistical power, particularly in subtype contrasts and multivariable models. HLA typing was restricted to DRB1, DRB3/DRB4/DRB5 and inferred DRB1-DQB1 haplotypes, without data on class I loci or genome-wide polygenic risk, and no functional immune, infection or inflammatory markers were collected, so mechanistic interpretations remain plausible but speculative rather than causal.

Cannabis exposure was defined clinically, with participants identified as regular users (at least weekly), but standardized quantitative information on dose, cumulative duration and age at first use was not available. This prevents dose–response analyses and introduces heterogeneity within the cannabis-user groups, limiting the precision with which cannabis-related risk can be characterized. Collapsing DRB1/DQB1 into allele-group markers and coding DRB3/DRB4/DRB5 as presence/absence indicators also reduces immunogenetic resolution in this highly polymorphic region, so the present findings are best viewed as signals at the level of broader HLA class II backgrounds that require confirmation in larger, more deeply typed cohorts.

Finally, we did not collect ancestry-informative markers or perform formal ancestry adjustment, and multivariable models included only a limited set of HLA markers without comprehensive covariate control. Residual confounding by demographic, clinical and environmental factors, as well as population stratification, therefore remains a substantial possibility, and the observed HLA–psychosis associations should be interpreted as preliminary and hypothesis-generating rather than as evidence of definitive allele-specific or mechanistic effects.

In particular, markers that only reached trend-level significance (0.05 ≤ *p* < 0.15) should be regarded strictly as hypothesis-generating, as they are highly susceptible to sampling fluctuation even after FDR correction.

Nevertheless, the convergence of Fisher tests with multiple-testing correction, resampling evidence for DRB5 and consistent patterns across clinically meaningful comparisons suggests that certain HLA-DRB5/DRB1*16 and DRB4/DRB3 class II backgrounds may modify vulnerability to psychosis in cannabis users, particularly with respect to schizophrenia versus other psychotic outcomes, rather than determining risk on their own. Given the cross-sectional, observational design and the absence of direct immunological measurements, these patterns should be interpreted as indicative of immunogenetic modifiers within a broader, multifactorial risk architecture, and not as evidence for specific causal immune mechanisms.

At the same time, several markers outside the core DRB1*16/DRB5/DRB4-DRB3 background showed only weak or trend-level associations after FDR correction, with wide confidence intervals and limited robustness in sensitivity analyses. These findings should be interpreted with particular caution and viewed primarily as a basis for hypotheses to be tested in larger, adequately powered studies, rather than as firm evidence of additional HLA effects in cannabis-exposed populations.

More broadly, the study was conducted in a single, moderately sized clinical cohort without detailed, standardized measures of cannabis dose, potency, or age at first use, and our genotyping was restricted to selected class II loci rather than the full MHC region. These constraints, together with the absence of direct immune or infectious biomarkers, mean that our data cannot establish causal mechanisms and may not generalize to other populations or treatment settings. Within these limitations, the patterns observed here offer a starting point for future work integrating HLA class II profiling with refined exposure metrics and immunological or neurobiological readouts, which is further outlined in the Conclusions Section 5.

## 5. Conclusions

In this cohort of individuals with a history of cannabis use, HLA class II profiles were linked to distinct psychotic outcomes, with DRB5/DRB1*16-containing backgrounds associated with higher odds of psychotic disorders and DRB4/DRB3 paralogs associated with lower odds of schizophrenia within the exposed group. These patterns support the idea that immunogenetic background can modify how cannabis-related risk translates into specific clinical diagnoses, complementing established dopaminergic and environmental contributors. Because the analyses were conducted in a single, moderately sized sample and several signals were modest or trend-level, the results should be viewed as preliminary and as a starting point for replication and refinement in larger, independent cohorts. Future studies that combine detailed characterization of cannabis exposure with broader HLA and complement typing, immune and infectious markers, and neurobiological readouts may clarify the mechanisms by which HLA class II variation shapes vulnerability and resilience to cannabis-related psychotic disorders.

## Figures and Tables

**Table 1:** Sociodemographic and socio-familial profile of cannabis users without psychosis, with non-schizophrenia psychosis, and with schizophrenia (lots 0, 1 and 2), including age, sex, area of residence, marital status, parental migration, living situation, education and occupation.

Characteristic	Category	Lot 0: CU No Psychosis	Lot 1: CU + Non-Schizophrenia Psychosis	Lot 2: CU + Schizophrenia	Total
Sex	Male	178/80.90%	36/85.71%	28/77.78%	242/81.11%
	Female	42/19.1%	6/14.29%	8/22.22%	56/18.79%
Age (years)	Mean ± SD	26.47 ± 5.79	25.76 ± 5.64	26.36 ± 4.49	26.41 ± 5.60
	Range	18–47	19–47	20–38	18–47
Area of residence	Urban	183/76.89%	30/71.43%	28/77.78%	241/74.66%
	Rural	55/23.10%	12/28.57%	8/22.22%	75/25.34%
Marital status	Married/cohabiting	55/25.23%	6/14.29%	6/16.67%	67/25.34%
	Single (never married)	150/68.81%	33/78.57%	28/77.78%	211/71.28%
	Divorced/widowed	13/5.96%	3/7.14%	2/5.56%	18/6.08%
Parents’ marital status	Married/cohabiting	130/59.63%	28/66.67%	24/66.67%	182/61.49%
	Single (never married)	6/2.75%	2/4.76%	1/2.78%	9/3.04%
	Divorced/widowed	82/37.61%	12/28.57%	11/30.56%	105/35.47%
Parents’ migration	No	165/80.49%	36/92.31%	30/88.24%	231/83.09%
	Yes	40/19.51%	3/7.69%	4/11.76%	47/16.91%
Living situation	With family	131/60.09%	26/61.90%	17/47.22%	174/58.78%
	With partner/friends	23/10.55%	3/7.14%	6/16.67%	32/10.18%
	Alone/unstable	64/29.36%	13/30.95%	13/36.11%	90/30.41%
Education	≤8 years	33/15.14%	4/9.52%	3/8.33%	40/13.51%
	High school	152/69.72%	31/73.81%	21/58.33%	204/68.92%
	University or higher	33/15.14%	7/16.67%	12/33.33%	52/17.57%
Occupation	Employed	66/30.28%	9/21.43%	9/25%	84/28.38%
	Student	26/11.93%	3/7.14%	6/16.67%	35/11.82%
	Unemployed/no job	74/33.94%	21/50%	13/36.11%	108/36.94%
	Retired/other income	52/23.85%	9/21.43%	8/22.22%	69/23.31%
Religion	Religious	86/39.45%	23/54.76%	13/36.11%	168/56.76%
	Not religious	132/60.55%	19/45.24%	23/63.89%	128/43.24%

**Table 2:** Universal association between HLA-DRB1 alleles, DRB3/DRB4/DRB5 paralogs and the DRB1*16-DQB1*05 haplotype and the presence of psychotic disorders among cannabis users, comparing cannabis users without psychosis (lot 0) to those with any psychosis (lots 1+2), with odds ratios, 95% confidence intervals, *p*-values and FDR-adjusted q-values.

Marker	OR	Lower CI	Upper CI	*p*-Value	q FDR
DRB1*16	2.73	1.47	5.07	<0.01	0.01
DRB5	2.25	1.35	3.77	<0.01	0.01
DRB1*16-DQB1*05	3.03	1.20	7.63	0.02	0.04
DRB1*07	0.35	0.12	0.98	0.04	0.06
DRB4	0.60	0.33	1.08	0.09	0.11
DRB3	0.65	0.37	1.14	0.13	0.13

**Table 3:** Association of selected HLA class II markers with non-schizophrenia psychosis in cannabis-using patients, comparing cannabis users without psychosis (lot 0) to cannabis users with non-schizophrenia psychotic disorders (lot 1), with odds ratios, 95% confidence intervals, *p*-values and FDR-adjusted q-values.

Marker	OR	Lower CI	Upper CI	*p*-Value	q FDR
DRB1*16	2.26	1.07	4.79	0.03	0.03
DRB1*15-DQB1*06	0.27	0.04	1.68	0.14	0.14

**Table 4:** Association of HLA-DRB1 alleles and DRB3/DRB4/DRB5 paralogs with schizophrenia among cannabis users, comparing cannabis users without psychosis (lot 0) to cannabis users with schizophrenia (lot 2), with odds ratios, 95% confidence intervals, *p*-values and FDR-adjusted q-values.

Marker	OR	Lower CI	Upper CI	*p*-Value	q FDR
DRB1*03	4.11	0.77	21.94	0.12	0.12
DRB4	0.15	0.04	0.59	<0.01	<0.01
DRB5	2.50	1.09	5.69	0.04	0.04

**Table 5:** Differential HLA class II associations between schizophrenia and other psychotic disorders among cannabis users, comparing cannabis users with non-schizophrenia psychosis (lot 1) to those with schizophrenia (lot 2), with odds ratios, 95% confidence intervals, *p*-values and FDR-adjusted q-values.

Marker	OR	Lower CI	Upper CI	*p*-Value	q FDR
DRB1*16	0.27	0.09	0.83	0.02	0.07
DRB1*08	8.93	0.42	192.19	0.14	0.14
DRB1*03	4.23	0.59	30.19	0.14	0.14
DRB4	0.18	0.04	0.75	0.01	0.01

## Data Availability

The data are available from the corresponding author upon reasonable request, subject to ethical and privacy restrictions related to genetic and clinical information.
